# Stability and bidirectional relationship between physical activity and sedentary behaviours in Brazilian adolescents: Longitudinal findings from a school cohort study

**DOI:** 10.1371/journal.pone.0211470

**Published:** 2019-01-25

**Authors:** Viviane S. Straatmann, Ylva B. Almquist, Aldair J. Oliveira, Gloria V. Veiga, Mikael Rostila, Claudia S. Lopes

**Affiliations:** 1 Department of Public Health and Policy, University of Liverpool, Liverpool, United Kingdom; 2 Aging Research Center, Karolinska Institute, Stockholm, Sweden; 3 Department of Public Health Sciences, Stockholm University, Stockholm, Sweden; 4 Laboratory of Social Dimensions Applied to Physical Activity and Sport (LABSAFE), UFRRJ, Seropédica, Brazil; 5 Institute of Nutrition Josue de Castro, Federal University of Rio de Janeiro (UFRJ), Rio de Janeiro, Brazil; 6 Institute of Social Medicine, State University of Rio de Janeiro (UERJ), Rio de Janeiro, Brazil; Universite Cote d’Azur, FRANCE

## Abstract

**Purpose:**

We investigated the stability, correlations and bidirectional relationship of light physical activities (PA), moderate and vigorous PA (MVPA), television viewing (TV) and video game/computer use (VG) in Brazilian adolescents.

**Methods:**

Adolescent Nutritional Assessment Longitudinal Study-ELANA is a middle school cohort study conducted in Rio de Janeiro-Brazil in 2010–2012. Self-reported data on PA (International Physical Activity Questionnaire- IPAQ) and screen activities were obtained from 810 adolescents (mean ages of 10.9 years old (SD 0.78) for girls; 11 years old (SD 0.85) for boys) to perform autoregressive cross-lagged structural equation models in two time points for PA and three time points for screen activities.

**Results:**

There was no significant stability of light PA and MVPA for boys and girls. Moderate stability of screen activities were found for both genders, with a significant coefficient of TV for boys (T1-T2:0.29; T2-T3:0.27 p<0.001); and VG for boys (T1-T2:0.33; T2-T3:0.35 p<0.001) and girls (T1-T2: 0.26; T2-T3:0.37 p<0.01). Significant lagged effects were obtained only among girls: light PA had effect on VG (-0.10 p<0.01), as well as in the opposite direction of TV on light PA (-0.03 p<0.01) and TV on MVPA (-0.11 p<0.01).

**Conclusion:**

The light PA, MVPA and screen activities (among girls) did not demonstrate stability over time. A warning scenario was suggested by the stability of high amounts of screen activities among boys over time. Screen activities had bidirectional association with light PA and MVPA among girls over time.

## Introduction

Physical inactivity is as a global pandemic that causes morbidity, mortality [1] and substantial economic burden [2]. Worldwide, only one-fifth of youths are estimated to be sufficiently physically active, whereas sedentary behaviours are even more predominant on the daily time of children and adolescents [3, 4]. For adolescents, the health benefits of physical activity (PA) in higher levels of intensity (moderate and vigorous PA [MVPA]) are well-established in the literature [5]. Additionally, a growing body of evidences have been shown that light-intensity of PA (e.g. walking) may provide important health benefits [6–8]. On the other hand, high levels of sedentary behaviours have been associated with obesity and metabolic risk factors in children and adolescents [9–11], and to early mortality in adulthood [12].

The engagement in daily activities varies across the lifespan, being particularly affected by transition phases in life [13–15]. However, if positive health behaviours such as regular practice of PA and low amount of time spend with sedentary activities (e.g. television viewing [TV] or video games [VG]) are initiated early in life, there is greater chance of continuing into later in life [15]. Thus, the tendency of individuals to maintain their behaviours over time is known as stability [16]. PA seems to be less stable in early childhood and in transition phases (e.g. from childhood to adolescence, and from adolescence to adulthood) [14, 15]. On the other hand, sedentary behaviours such as screen activities, which have a strong habitual element among adolescents, are likely to be relatively stable over time [14, 17].

PA and sedentary behaviours are distinct constructs which potentially influence each other, and possible to co-exist without detriment [18, 19]. A systematic review with meta-analyses of the relationship between these behaviours, suggested that the mere idea of displacement among PA and sedentary behaviours (e.g. a person with high levels of PA must not be, consequently, sedentary, and vice versa) cannot be the only source of explanation to be addressed. The authors identified a small and negative association between PA and sedentary behaviours, and also indication that a much more complex setting involving other factors should to be considered [19].

Thus, evaluating stability and changes over time of PA and sedentary behaviours can be useful to better understand patterns of health behaviour across the adolescence. In fact, previous studies showed a decline of PA throughout adolescence. Rauner et al. (2015) [20], using a representative sample of Germany adolescents, found low stability in both genders. A recent study that was conducted in Sweden [21], also showed a decrease of PA across adolescence. Although the literature is vast, few studies have focused on the beginning of adolescence. Moreover, to the best of our knowledge, there are no studies that have investigated the possible associations between PA and sedentary behaviours in longitudinal data, using a robust statistics procedure (e.g. structural equation modelling). Arguably, more in-depth knowledge about the relationship between PA and sedentary behaviours as well as the direction of associations (e.g. PA affects screen activities or screen activities affect PA over time), can help to create appropriate interventions and health policies in order to promote healthy life style among youths to be carried throughout life. Furthermore, most of evidence in this area come from developed countries and do not reflect the specific socioeconomic circumstances of low- and middle- income countries.

There is evidence suggesting that PA and sedentary behaviours might vary by gender [22]. Girls often report more screen time and less PA compared with boys. Therefore, considering the genders separately may be a better approach to investigate the stability of PA and screen activities as well as the possible bidirectional associations.

We aimed to investigative the following points in a middle school cohort of Brazilian’s boys and girls from 2010 to 2012: (a) to examine the stability of PA (light PA and MVPA) and screen activities (TV viewing and computer/ video games use [VG]); (b) to examine cross-sectional and bidirectional associations, longitudinally, between PA and screen activities.

## Materials and methods

### Study design

The Adolescent Nutritional Assessment Longitudinal Study (ELANA) followed two cohorts (middle and high school) at two public and four private schools from the metropolitan area of Rio de Janeiro, Brazil. The metropolitan area of Rio de Janeiro is formed by 18 municipalities representing the second largest centre of national wealth in the country [23]. In contrast, the geographic area of ELANA’s schools faces many social disparities which results in a lack of security and vulnerable neighbourhoods and poverty [24]. The main aim of the ELANA was to examine changes in anthropometric indicators and body composition, and to verify the influence of health, socioeconomic and psychosocial factors on inadequate development and nutritional status.

This study included data from the middle school cohort from 2010 to 2012. At baseline, a self-reported questionnaire was administered to investigate physical activity, sedentary behaviour, socioeconomic and demographic variables among the adolescents. Questionnaire of physical activity was repeated in 2011, and sedentary behaviours in 2011 and 2012.

This research was conducted according to the guidelines established in the Declaration of Helsinki. The Ethics Committee of Research of the Institute of Social Medicine of the State University of Rio de Janeiro (certificate number 0020.0.259.000–09) approved all procedures involving human subjects before the start of the study. Adolescents’ legal guardians provided a written informed consent. All the data we used were confidential and the researchers did not have access to any personal data that could identify individuals included in the study. Following the internal regulations of the ELANA Study committee, data are made available for specific research projects. Thus, we are not allowed to share the data we used for this study with other researchers. However, we are glad to answer questions about the data used in this study and to share unpublished results.

### Study sample

Out of the 946 adolescents available, 888 met the eligibility criteria for this study, which included not having a physical or mental condition preventing the completion of questionnaires and/or not being pregnant or lactating at the time of data collection. Of the 888 eligible adolescents, 32 (3.6%) refused to participate, 46 (5.2%) did not have parental consent, and 4 (0.45%) had no birth date information. The study sample comprised 810 students at baseline, of which 786 adolescents completed information of physical activity and sedentary behaviours at baseline, corresponding to a response rate of 88.5%. In 2011, 526 students repeated the evaluation of physical activity and sedentary behaviours (33% missing cases), and 435 of sedentary behaviours in 2012 (46% missing cases). Imputation of missing cases was carried out by means of maximum likelihood estimation, allowing the study to include 810 adolescents in all time points.

### Measurements

#### Physical activity (PA)

Physical activity (PA) was assessed by the short version of the self-reported ‘International Physical Activity Questionnaire–IPAQ’ which has eight open questions estimating the weekly time of vary intensities of PA during the last 7 days (light PA: walking; moderate PA: carrying light loads, bicycling at a regular pace, or doubles tennis; and vigorous PA: lifting, digging, aerobics, or fast bicycling). This questionnaire was validated by Guedes et al. (2005) [25] for Brazilian adolescents older than 14 years old. The Brazilian version of short IPAQ has been validated against a 24-hour recall instrument of the daily activities, identifying correlation coefficients varying between 0.49 to 0.83. When detecting blank answers and unusual values for responses (e.g. the adolescent said they performed eight hours of MVPA per day) the research staff returned to the student in order to check the coherence of the answer, avoiding loss of information and classification errors. The time (in minutes) of light PA and MVPA per day was used, and it was based on the guidelines for data processing and analyses of the IPAQ [26].

#### Sedentary behaviours (screen activities)

The television viewing (TV) and video game/computer use (VG) times were assessed separately by two questions from a self-reported questionnaire [27]. The first question was ‘*How many days do you watch TV and use VG per week*? (1) Almost never or never, (2) 1 to 2 times per week, (3) 3 to 4 times per week, (4) 5 to 6 times per week, and (5) every day’. The responses were categorised in a five-point scale: category 1 = 0 days (almost never or never), 2 = 1.5 days, 3 = 3.5 days, 4 = 5.5 days, 5 = 7 days. The second question was *‘In general*, *how many hours do you usually spend watching TV and using VG per day*?*’*. Average daily time in minutes was calculated multiplying ‘hours per day’ by ‘days per week’ for TV and VG variables, applying the formula: [(days per week)*(hours per day)]*60/7.

#### Covariates

Age, Asset ownership, body mass index (BMI) and sexual maturation were treat as potential baseline confounders of the investigated association. The economic characteristics of the families of the adolescents were assessed through the Brazilian Socioeconomic Classification Criteria (CCEB) [28], which takes into account the purchasing power of urban households based on a score obtained by the sum of the household assets, the presence of domestic workers in the household and the schooling level of the head of household. An indicator constructed from information on ownership of durable assets in the home was used to calculate the presence or absence of, respectively: TV; VCR or DVD player; radio; bathroom; automobile; washing machine; refrigerator; and freezer (independent appliance or part of duplex refrigerator). A weighted score was attributed considering presence and relative frequency of each item [29].

The Body mass index (BMI in kg/m^2^) was calculated based on direct measurements of height and weight [30]. Sexual maturation stage was investigated using the self-evaluation technique validated by Saito (1984) [31], focusing on the development of genitalia for boys and breast for girls, according to Tanner’s (1962) [32] criteria. This information was coded as a categorical variable (pre-pubescent, early spurt, maximum speed peak, and slowing growth).

### Statistical analyses

Firstly, the distribution of all variables in the sample was tested by the Kolmogorov Smirnov test. Comparisons between the genders were performed through the non-parametric Wilcoxon Test, and Wilcoxon Rank Compared Test to evaluate differences between means ranks over time from T1 to T2. In order to facilitate the interpretation of the descriptive results, we presented means, standard deviations, minimum and maximum, previously tested and that showed similar statistical results with non-parametric tests (p values) (details available upon request).

Due to the non-normal distribution of the sample, an asymptotic estimation (more appropriate for non-parametric distributions, without missing value imputation) was performed instead of the maximum likelihood missing values estimation. All coefficient values remained similar in this analysis, but lost their statistical significance, most likely due to the decreased sample size. Hence, it was decided to continue the analysis with the maximum likelihood for missing values estimation. The maximum likelihood estimation was used to deal with missing values, which is probably the most pragmatic missing data estimation approach for structural equation modelling (SEM), and has been shown to produce unbiased parameter estimates and standard errors for data missing at random and data missing completely at random [33]. School changes by students are very common in Brazil, making it difficult to follow them, and these are probably not related to the outcomes or the other variables being investigated. Further sensitivity analysis was performed to support the assumption of missing at random or completely random for the imputation process (available upon request).

**Fig 1:**
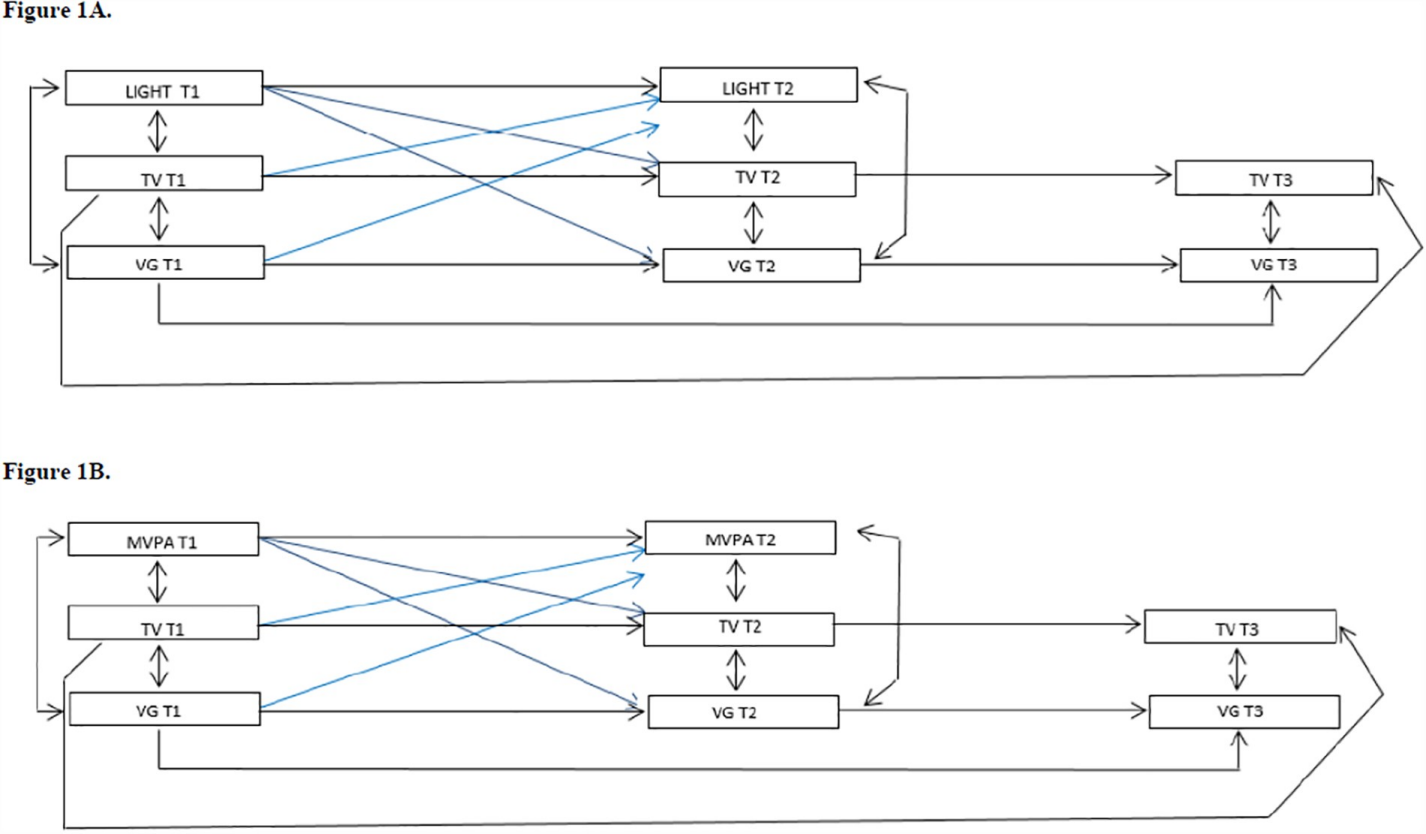
Logic models. (1a) Model A for light physical activity (PA); (1b) Model B for moderate and vigorous physical activity (MVPA). The Adolescent Nutritional Assessment Longitudinal Study, 2010–2012, Brazil.

Autoregressive cross-lagged panel models were estimated by means of SEM to simultaneously address bidirectional association of PA and screen activities, with maximum likelihood missing values estimation. Two different models were performed, considering the intensities of PA [Model A: light PA; Model B: MVPA, both stratified by gender. A baseline model was constructed with autoregressive paths (measuring stability over time) from light PA and MVPA (Models A and B, respectively) at Time 1 (T1-2010) to Time 2 (T2-2011), and from screen activities (TV and VG) at T1 to T2, and from T2 to Time 3 (T3-2012). The autoregressive model also included correlations between PA (light PA: model A and MVPA: model B) and screen activities at T1 and T2; and within screen activities (TV and VG) at T3. Apart from the baseline model, three additional models were assessed which included one or more cross-lagged estimate (I. Forward causation model: PA at T1 predicts screen activities at T2; II. Reversed causation model: screen activities at T1 predict PA at T2; III. Reciprocal model: PA and screen activities have reciprocal effects). Baseline confounders were included in all models but not shown in illustrations. The models were illustrated in Fig 1 (1a [Model A] and 1b [Model B]).

**Fig 2:**
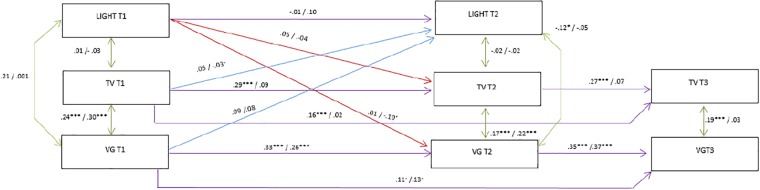
Associations between light activity, TV and VG (Males n = 436, Females n = 374). Results from structural equation modelling. Estimates (standardized) are displayed as males/females. *** p<0.001, ** p<0.01, * p<0.05(baseline confounders were omitted) The Adolescent Nutritional Assessment Longitudinal Study, 2010–2012, Brazil.

**Fig 3:**
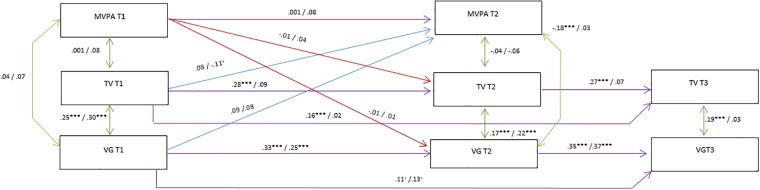
Associations between MVPA, TV and VG (Males n = 436, Females n = 374). Results from structural equation modelling. Estimates (standardized) are displayed as males/females. *** p<0.001, ** p<0.01, * p<0.05 (baseline confounders were omitted). The Adolescent Nutritional Assessment Longitudinal Study, 2010–2012, Brazil.

For each of the models (A and B), a set of model fit statistics was derived: the Standardised Root Mean Square Residual (RMSEA), which should be below or close to 0.06, as well as the Comparative Fit Index (CFI) and the Tucker-Lewis Index (TLI), which should both be close to or above 0.95. The p value for chi-square test was also presented for the model fit statistics, which should be above 0.05. Age, BMI, asset ownership and sexual maturation were used in the models as confounding variables assessed at baseline (suppressed on the Fig 1a and 1b [methods], and Figs 2 and 3 [results]. All analyses were performed in Stata version 13.1 (Stata Corp, Texas, USA).

## Results

Table 1 presents descriptive statistics for all of the variables. The mean age of the girls was 10.9 years old (SD 0.78), and 11 years old (SD 0.85) for boys. Gender differences were tested, showing that boys spend a significantly higher amount of time on light PA (T1: 27.7 minutes vs 24.6 minutes; T2: 27.2 minutes vs 19.2 minutes) and MVPA (T1: 67.9 minutes vs 42.9 minutes; T2: 41.3 minutes vs 29.1 minutes) compared with girls at both T1 and T2. At T2 girls spend significantly more time with VG than boys (181.6 minutes vs 166.6 minutes). There was no statistically significant gender difference for the remaining items. With regard to changes over time (from T1 to T2), the results showed a statistically significant increase in time spent on VG (boys: 142.1 minutes to 192.7 minutes; girls: 104.4 minutes to 181.6 minutes) and decrease in time spent on MVPA (boys: 67.9 minutes to 41.3 minutes; girls: 42.94 minutes to 29.12 minutes). There was no significant statistical difference on screen activities between T2 and T3 for boys or girls.

**Table 1:** Characterization and distribution of the study variables. The Adolescent Nutritional Assessment Longitudinal Study, 2010–2012, Brazil.

	N		Males				N		Females				Comparison males-females ^a^	
		%	Min	Max	Mean	St. dev.		%	Min	Max	Mean	St. dev.	Mean diff.	T-test
**Time 1**														
MVPA (minutes/day	431	-	0.0	360.0	67.9	65.3	370	-	0.0	240.0	42.9	43.4	23.0	***
Light Activity (minutes/day)	431	-	0.0	180.0	27.7	34.2	370	-	0.0	180.0	24.6	28.7	3.1	***
TV (minutes/day)	432	-	0.0	540.0	195.0	158.1	371	-	0.0	540.0	200.4	172.6	5.3	n.s.
VG (minutes/day)	435	-	0.0	540.0	142.1	146.0	371	-	0.0	540.0	104.4	133.1	9.0	***
BMI (kg/m^2^)	427	-	12.8	39.8	20.4	4.3	365	-	12.6	38.9	20.1	4.30	0.32	n.s.
Assets Indicator (%)	427						365							
Less asset ownership		25.3	-	-	-	-		12.7	-	-	-	-		
More asset ownership		74.7	-	-	-	-		87.3	-	-	-	-		
Sexual Maturation (%)	427						365							
Pre-pubescent		5.8	-					6.4	-	-	-	-		
Early spurt		73.1	-	-	-	-		28.9	-	-	-	-		
Maximum speed peak		16.3	-	-	-	-		47.0	-	-	-	-		
Slowing growth		4.4	-	-	-	-		17.7	-	-	-	-		
**Time 2**														
MVPA (minutes/day)	288		0.0	231.4	41.3	41.2	259		0.0	300.0	29.1	37.8	12.1	***
Light Activity (minutes/day)	288		0.0	154.3	27.2	31.8	259		0.0	180.0	19.2	26.6	8.1	***
TV (minutes/day)	288		0.0	594.0	192.7	159.0	259		0.0	594.0	193.5	150.0	-0.87	n.s.
VG (minutes/day)	288		0.0	540.0	166.6	163.0	254		0.0	540.0	181.6	176.8	-15.0	***
**Time 3**														
TV (minutes/day)	231		0.0	540.0	179.2	148.8	205		0.0	540.0	177.5	343.5	1.7	n.s.
VG (minutes/day)	230		0.0	540.0	168.8	156.1	205		0.0	540.0	161.2	150.6	7.7	n.s.
			**Mean diff**.		**T-test**				**Mean diff**.		**T-test**			
**Comparison T1-T2** ^b^														
MVPA (minutes/day)			-26.7		***				-13.8		***			
Light Activity (minutes/day)			-0.4		n.s				-5.4		n.s			
TV (minutes/day)			-2.3		n.s.				-6.8		n.s.			
VG (minutes/day)			24.5		***				77.2		***			
**Comparison T2-T3** ^b^														
TV (minutes/day)			-19.6		n.s.				-9.8		n.s.			
VG (minutes/day)			7.1		n.s.				-14.5		n.s.			

*** p<0.001,

** p<0.01,

* p<0.05.

^a^ A positive difference value reflects that males are higher off compared to females, whereas a negative difference value suggests the opposite.

^*b*^ A positive difference value indicates higher values over time, whereas a negative difference value reflects the opposite.

Satisfactory parameters of goodness-of fit statistics were presented in Table 2 for the models A (light PA) (RMSEA 0.025; CFI 0.944; TLI 0.910; p 0.115) and B (MVPA) (RMSEA 0.023; CFI 0.951; TLI 0.921; p 0.136). Overall, Models A and B presented similar results regarding stationary autoregressive effect and correlations of screen activities (TV and VG).

**Table 2:** Goodness-of fit statistics for the tested models (n = 810). The Adolescent Nutritional Assessment Longitudinal Study, 2010–2012, Brazil.

Model A- Light Activity*	
RMSEA	0.025
CFI	0.944
TLI	0.910
*χ*^2^	62.25
*Df*	50
*P*	0.115
Model B- MVPA*	
RMSEA	0.023
CFI	0.951
TLI	0.921
*χ*^2^	61.05
*Df*	50
*P*	0.136

*Baseline model versus saturated test. The saturated model is the model that fits the covariance perfectly, in our case, the reciprocal model. RMSEA- Standardized Root Mean Square Residual; CFI- Comparative Fit Index; TLI- Tucker-Lewis Index.

Regarding stability of light PA, there was no significant stationary autoregressive effect for both genders [boys (-0.01); girls (0.10)] (Fig 2). In Fig 3, it is shown that there was no stationary autoregressive effect of MVPA for both genders. Our findings showed that the stationary autoregressive effect of TV was significant for boys [T1-T2 (0.29), T2-T3 (0.27), p < 0.001]; the stationary autoregressive effect of VG was significant for boys [T1-T2 (0.33), T2-T3 (0.35), p<0.001] and girls [T1-T2 (0.26), T2-T3 (0.37), p < 0.01)]. These coefficients indicate low to moderate stability of screen activities over time (Figs 2 and 3).

Significant negative correlation between light PA and VG at T2 was observed among boys (-0.12, p<0.01) (Fig 2). At T2, MVPA was also negatively correlated to VG in boys ([(-0.18), p < 0.001] (Fig 3). Screen activities showed significant correlations between them in all time point for boys [T1 (0.24), p < 0.001; T2 (0.17), p < 0.001; T3 (0.19, p<0.001] and for girls at T1 and T2 [T1 (0.30), p < 0.001; T2 [(0.22), p < 0.001] (Figs 2 and 3).

Results regarding the bidirectional relationship of PA and screen time activities showed a significant lagged effect of light PA on VG in girls (-0.10, p < 0.01). There was also evidence of lagged effect in the opposite direction of TV on light PA also in girls (-0.03, p < 0.01) (Fig 2). In model B, lagged TV was negatively associated with MVPA (-0.11, p < 0.01), meaning that more time spent on TV at T1 was associated with less with MVPA at T2 among girls (Fig 3).

## Discussion

This study investigated the stability, correlations, and bidirectional relationships of light PA and MVPA with screen activities (TV viewing and VG use) in a cohort of Brazilian adolescents attending middle school. Our results demonstrated a significant increase of VG use and decrease of MVPA over time. Screen activities showed low to moderate stability among boys. A positive correlation between TV and VG was evident, as well as a negative correlation between both PA intensities and VG among boys, when they start getting older (T2). Bidirectional relationships were observed only among girls, for whom TV viewing had a negative influence on light PA and MVPA, whereas light PA was associated with higher VG use over time.

Similar patterns of increased VG use and decrease of TV viewing have been observed in adolescents from 30 developed countries, reported by Bucksch and colleagues (2016) [34]. VG use is gaining popularity as a secular trend at all ages due to targeting and availability of this technology to adolescents [35]. In Brazil, the early 2000s were characterised by positive social policies outputs of reducing economic disadvantages, which led to an increase in purchasing power and acquisition of technologies, such as computer and videogames, in Brazilian households [36, 37].

Our findings also demonstrated a low to moderate stability of screen activities coefficients in boys, partially consistent with results from a systematic review of stability of sedentary behaviours in young people [17]. Biddle and colleagues (2010) [17] demonstrated moderate stability of sedentary behaviours without significant differences between genders, and also that the stability of such coefficients for short time periods were larger than coefficients of long time periods. The absence of stability of both intensities of PA could be a repercussion of the significant decrease in the levels of this behaviour, as observed by Jones and colleagues (2013) [14] and Tammelin and colleagues (2014) [15]. These findings may be analysed in some perspectives. Based on social relationships background, adolescence is influenced by different relationships (e.g. parents, siblings, friends, and school colleagues) which play an important role in PA and sedentary behaviours [38]. Taking into account this wide spectrum of connections, the role of parents may change across adolescence, commonly providing more autonomy for them, especially with regard to their leisure routine. In this sense, the changes in social relationships throughout mid adolescence might be an explanation for the observed absence of stability. In addition, the role of self-efficacy, self-perception capability of engaging on or maintaining PA, as a mediator of the relationship between social support and PA has been demonstrated in the literature [39, 40]. It is plausible to postulate that levels of self-efficacy could change during adolescence, which in turn, would be related to unstable levels of PA.

This study reported a small and negative correlation between both intensities of PA and VG use in boys. Corroborating this, results from 10 year-old Brazilian children in the International Study of Childhood Obesity, Lifestyle and the Environment (ISCOLE) reported a negative relationship between MVPA with screen activity evaluated by time of TV viewing [41]. Melkevik et al. (2010) [42] also showed that, for both boys and girls from the Health Behaviour in School-aged Children (HBSC) study, viewing TV > 2 hours/day was significantly associated with accumulating less daily time in MVPA. However, the cross-sectional nature of these studies did not support further discussions related to the existence of a direct substitution effect of these behaviours, and either not allowed causal conclusions.

In line with previous studies [22], our findings revealed gender differences in both behaviours, PA and screen ones. Regarding the bidirectional relationship analysis, it was only among girls that the results demonstrated an association between light PA and VG use. Moreover, the results showed a negative association between TV viewing and MVPA. These findings could be analysed in light of the displacement theory, which suggests that TV viewing displaces time that could be spent in other more active pursuits. Although these results did not showed a total displacement across activities, it is plausible that girls had a greater risk of switching from PA to screen behaviours, as compared to boys. In this sense, gender differences in the stability of sedentary behaviours could also, at least partially, be explained by displacement reported.

Alongside of this literature, there was evidence from experimental research suggesting that the association between sedentary behaviour and PA may be asymmetrical, such that modification of children’s sedentary behaviour would impact upon PA just under particular conditions [43, 44]. Pearson and colleagues (2014) [19], based on findings from a systematic review and meta-analysis, suggested that when developing interventions to promote PA, strategies targeting reduction in sedentary behaviours may be beneficial only when employed as part of a broader package of measures targeting the determinants of PA [19].

In a longitudinal study (4 year-follow-up) of children from the US, Taveras et al. (2007) [45] found that changes in TV viewing were not associated with corresponding changes in time spent on MVPA. On the other hand, results from Iowa Bone Development Study (IBDS) cohort showed that a consistently active trajectory was associated with decreasing in an already low TV viewing trajectory for both boys and girls [46]. Our findings showed that a high amount of TV viewing was associated with less time spent on light PA and MVPA one year later, only among females. This finding may suggest adverse consequences for health, since there is evidence of the influence of high amounts of screen activities on weight (obesity) [47] and worse psychological well-being in girls [48].

### Strengths, limitations and implications

To the authors’ knowledge, the present study is the first to use a cross-sectional and longitudinal approach with robust analytical framework to demonstrate weak stability of light PA and screen activities in girls and a moderate stability of MVPA in boys, and also, a bidirectional association between PA and screen ones among girls in a Latin American adolescent population. The analysis strategy was one of strengths of this study, which minimized the possibility of biases. Furthermore, different screen activities were reported, making feasible discussions related to the type of technology accessed by Brazilian youths. The investigation of distinct intensities of PA was also a strength.

Limitations of our study, particularly related to its observational nature, include self-reported information about PA and screen activities, which request the ability of adolescents to remember, interpret, and quantify behaviours. It is also important to consider that the instrument used has been validated for adolescents of 14 years of age or older, which may have potentiated the mentioned problems regarding self-report of PA. Questions used to evaluate screen activated were based on the previous literature, but were not validated for this specific sample. In addition, might be important to pointed out that the screen behaviours questions did not include smartphones and other technology devices. The missing cases through the follow-up and the potential selection bias (greater participation in the second stage of adolescents with certain characteristics) should be mentioned as a potential limitation. However, it is also important to emphasize that data imputation methods (maximum likelihood estimation) were used, which probably minimized the possibility of bias. Furthermore, as with any type of non-randomized epidemiological research, the absence of confounders cannot be presumed, adjustments for possible confounding factors were made in order to minimise further bias. Despite the restriction of this study to a single geographical area, which makes generalizability limited, there is need to report evidences from specific context experienced by less affluent countries.

## Conclusion

The light PA and MVPA did not demonstrate stability over time suggesting, in this case, that adolescents decreased their PA over time. Our results also indicated the absence of stability of screen activities among girls, suggesting that the time spent with screen activities increased considerably. The stability of screen activity among boys is also worrying since it means that the elevated amount of time on screen activities initially assessed remains high over time. The health behaviours interacted more over time between girls, underscored by the potential influences of screen activities on PA.

The results reported here present a major public health concern. The consequences of unhealthy behaviours (e.g. hypertension, diabetes, obesity, and further chronic diseases) over the life course, result in staggering costs for health care systems. Interventions should be focused on reducing excessive sedentary behaviours and increase PA early in life. Besides this, there is need to address and understand the main reasons why adolescents give up of healthy behaviours. Future research may consider longer periods, covering all stages of adolescence, as well as the use of direct assessments of PA and sedentary (accelerometer) to obtain more reliable information of these constructs.
